# *E*-2-hexenal promotes susceptibility to *Pseudomonas syringae* by activating jasmonic acid pathways in *Arabidopsis*

**DOI:** 10.3389/fpls.2013.00074

**Published:** 2013-04-12

**Authors:** Alessandra Scala, Rossana Mirabella, Cynthia Mugo, Kenji Matsui, Michel A. Haring, Robert C. Schuurink

**Affiliations:** ^1^Department of Plant Physiology, Swammerdam Institute for Life Sciences, University of AmsterdamAmsterdam, Netherlands; ^2^Department of Biological Chemistry, Faculty of Agriculture, Graduate School of Medicine, Yamaguchi UniversityYamaguchi, Japan

**Keywords:** green leaf volatiles, *Pseudomonas syringae*, jasmonate, coronatine, hormone crosstalk

## Abstract

Green leaf volatiles (GLVs) are C6-molecules – alcohols, aldehydes, and esters – produced by plants upon herbivory or during pathogen infection. Exposure to this blend of volatiles induces defense-related responses in neighboring undamaged plants, thus assigning a role to GLVs in regulating plant defenses. Here we compared *Arabidopsis thaliana* ecotype Landsberg *erecta* (L*er*) with a *hydroperoxide lyase* line, *hpl1*, unable to synthesize GLVs, for susceptibility to *Pseudomonas syringae* pv. *tomato* (DC3000). We found that the growth of DC3000 was significantly reduced in the *hpl1* mutant. This phenomenon correlated with lower jasmonic acid (JA) levels and higher salicylic acid levels in the *hpl1* mutant. Furthermore, upon infection, the JA-responsive genes *VSP2* and *LEC* were only slightly or not induced, respectively, in *hpl1*. This suggests that the reduced growth of DC3000 in *hpl1* plants is due to the constraint of JA-dependent responses. Treatment of *hpl1* plants with *E*-2-hexenal, one of the more reactive GLVs, prior to infection with DC3000, resulted in increased growth of DC3000 in *hpl1*, thus complementing this mutant. Interestingly, the growth of DC3000 also increased in *Ler* plants treated with *E*-2-hexenal. This stronger growth was not dependent on the JA-signaling component MYC2, but on ORA59, an integrator of JA and ethylene signaling pathways, and on the production of coronatine by DC3000. GLVs may have multiple effects on plant–pathogen interactions, in this case reducing resistance to *Pseudomonas syringae* via JA and ORA59.

## INTRODUCTION

Plants produce green leaf volatiles (GLVs), C6-aldehydes, C6-alcohols, and their acetates, through the lipoxygenase (LOX) and hydroperoxide lyase (HPL) pathways. Linoleic and linolenic acid are the substrates for dioxygenation and subsequent cleavage to obtain C6-volatile aldehydes that can be further modified by alcohol dehydrogenases (ADH), an isomerization factor and an acetyltransferase leading to the formation of a bouquet of these volatiles. Intact plants produce only trace amounts of GLVs, whereas these compounds are rapidly emitted in large amounts after wounding, herbivory or pathogen attack (Croft et al., 1993; Turlings et al., 1995; Fall et al., 1999; Shiojiri et al., 2000, 2006a; Heiden et al., 2003).

Green leaf volatiles have been reported to play important roles in different biological processes (Bate and Rothstein, 1998; Arimura et al., 2000; Farag and Paré, 2002; Engelberth et al., 2004; Farag et al., 2005; Ruther and Fürstenau, 2005; Ruther and Kleier, 2005). Herbivory induces very specific sets of GLVs that are perceived by natural predators of the herbivores (Kessler and Baldwin, 2001; Birkett et al., 2003; Gouinguené et al., 2005; Shiojiri et al., 2006a,b). Beside a role in indirect defenses, GLVs also act as airborne signaling molecules regulating plant defense responses. Several studies show that plants themselves upon exposure to GLVs respond by activating wound- and herbivore-induced defenses. Examples of this are found in *Zea mays* (maize), *Citrus jambhiri*, *Nicotiana attenuata* (tobacco), *Gossypium hirsutum*, *Lycopersicon esculentum* (tomato), and *Arabidopsis thaliana* plants where GLV perception induces the transcription of genes known to be involved in defense responses, or in biosynthesis of defense-related secondary metabolites (Bate and Rothstein, 1998; Arimura et al., 2001; Gomi et al., 2003; Weber et al., 2004; Farag et al., 2005; Kishimoto et al., 2005, 2006; Paschold et al., 2006), resulting in the production of defensive compounds (Zeringue, 1992; Bate and Rothstein, 1998; Farag and Paré, 2002; Engelberth et al., 2004; Farag et al., 2005; Ruther and Fürstenau, 2005; Kishimoto et al., 2006; Yan and Wang, 2006). Besides direct defense elicitation, exposure to GLVs, emitted from wounded leaves, has also been shown to prime systemic leaves for augmented defense responses upon future attacks (Engelberth et al., 2004; Kessler et al., 2006; Frost et al., 2007, 2008; Heil and Silva Bueno, 2007). Similarly, the *E*-2-hexenal released by rice upon planthopper infestation, induces expression of defense-related genes, increasing resistance to bacterial blight (Gomi et al., 2010). In some of these examples the effect of GLVs and jasmonic acid (JA) signaling have been linked (Engelberth et al., 2004; Halitschke et al., 2004; Kishimoto et al., 2006; Allmann et al., 2010; Tong et al., 2012).

Finally, GLVs possess fungicidal and bactericidal activity (Prost et al., 2005; Shiojiri et al., 2006b). Since GLVs are released after infection with pathogenic fungi and bacteria (Croft et al., 1993; Heiden et al., 2003; Shiojiri et al., 2006b), this suggests that a possible physiological role of these volatiles is to limit pathogen growth. Several observations support this hypothesis. For instance, upon infection with the pathogenic bacteria *Pseudomonas syringae*, *Phaseolus vulgaris* (lima bean) leaves release relatively high amounts of the C6-aldehyde *E*-2-hexenal and the C6-alcohol Z-3-hexenol (Croft et al., 1993). Moreover, pre-treatment with the C6-aldehyde *E*-2-hexenal as well as genetic manipulation to enhance C6-volatile production, resulted in increased resistance against the necrotrophic fungus *Botrytis cinerea* in *Arabidopsis*, most likely as a result of both activation of defense responses and direct inhibition of fungal growth (Kishimoto et al., 2005; Shiojiri et al., 2006b).

Since all this evidence indicates a role for GLVs in regulating plant responses to bacterial pathogens and GLV levels have been shown to increase in plants upon infection with *Pseudomonas syringae* (Croft et al., 1993; Heiden et al., 2003), we decided to further dissect the role of GLVs in the interaction of plants with this pathogen. Increased GLV levels could directly inhibit the pathogen and/or promote infection through downstream signaling favorable for the pathogen. *Pseudomonas syringae* pv. *tomato* DC3000 is a plant pathogen that enters leaves through stomata, multiplies in the apoplast, and produces necrotic lesions with chlorotic halos (Hirano and Upper, 2000). *Pseudomonas syringae* pv. *tomato* DC3000 (DC3000) causes bacterial speck on tomato (Cuppels, 1986), but also on *A. thaliana *(Whalen et al., 1991). DC3000 produces coronatine (COR), a toxin, responsible for chlorotic halos, which mimics the action of JA-isoleucine (JA-Ile), the active form of JA. With this phytotoxin DC3000 exploits the antagonistic interaction between JA and salicylic acid (SA) in order to shut down SA-dependent defenses that plant triggers to fight against *Pseudomonas* infections (Block et al., 2005; Glazebrook, 2005).

We especially focused on the role of *E*-2-hexenal during the *Arabidopsis*–*Pseudomonas* interaction. Although it is not the most abundant C6-volatile produced by HPL activity, *E*-2-hexenal is emitted during *Pseudomonas* ssp. infections in lima bean (Croft et al., 1993) and in tobacco (Heiden et al., 2003), and it has the highest bactericidal activity *in vitro* among oxylipins (Prost et al., 2005), likely because its α,β-unsaturated carbonyl moiety that can react with nucleophilic groups (Farmer and Davoine, 2007). Additionally, *E*-2-hexenal has been shown to induce several responses in *Arabidopsis*, including induction of defenses, inhibition of root growth and enhancement of resistance against the necrotrophic fungus *B. cinerea* (Bate and Rothstein, 1998; Kishimoto et al., 2005; Mirabella et al., 2008). In order to determine the role of GLVs in the responses against *Pseudomonas*, we set out to study *Arabidopsis* plants with and without a functional HPL (Shiojiri et al., 2012) and did complementation studies with *E*-2-hexenal. Remarkably we found that the presence of a working copy of HPL increased susceptibility of *Arabidopsis* to DC3000. Treatment with *E*-2-hexenal also enhanced the susceptibility to this bacterial pathogen. We found evidence that this is mediated by the transcription factor ORA59, one of the main players in the JA-signaling pathways, and required the production of the bacterial toxin COR.

## MATERIALS AND METHODS

### PLANT LINES

*Arabidopsis thaliana* ecotype Columbia-0 (Col-0) and Landsberg *erecta* (L*er*) were used. The *hpl*1 mutant is an introgression line between Col-0 and L*er* (Shiojiri et al., 2012). The mutant *myc2* (*jin1-7*; Verhage et al., 2011), the transgenic lines RNAi-ORA59 and the 35S:GUS plants (Pré et al., 2008) were all in the Col-0 background. Plants were grown in soil in a growth chamber at 21°C, 70% relative humidity under an 11-h photoperiod with 100 μE s^-1^ m^-2^.

### BACTERIAL POPULATION COUNTS

Bacteria were grown overnight at 28°C in liquid King’s broth (KB) medium (King et al., 1954) containing rifampicin (50 μg/ml) for the *Pseudomonas syringae* pv. *tomato* DC3000 strain, and kanamycin (100 μg/ml) for the cor^-^ DC3682 mutant strain, unable to produce COR (Ma et al., 1991). Plants were inoculated with either a low dose (OD_600_ of 0.0007), for bacterial growth assays, or a high dose (OD_600_ of 0.007), for qRT-PCR and hormone quantification, of the bacterial suspension, and bacteria (colony forming units, cfu) were counted as reported in Park et al. (2010).

### PLANT HORMONES EXTRACTION AND QUANTIFICATION

For JA and SA quantification, 12 leaves were harvested, in pools of 4, from 12 different mock-infiltrated (10 mM MgSO_4_) or bacteria-infiltrated plants in two independent experiments. To extract JA and SA, frozen leaf material (50–150 mg) was ground and homogenized in 0.5 ml 70% methanol, spiked with 200 ng of D6-JA and D6-SA (internal standards for extraction efficiency; CDN Isotopes, Canada^1^), with a Precellys24 automated lyser (Bertin Technologies^2^). Samples were homogenized twice by shaking at 6,000 rpm for 40 s and centrifuged at 10,000 *g* for 20 min at 4°C. The supernatants of two extraction steps were pooled. Hormones were quantified by liquid chromatography–mass spectrometry (LC–MS) analysis on Varian 320 Triple Quad LC/MS/MS. Ten microliters of each sample were injected onto a C18 Pursuit 5 (50 mm × 2.0 mm) column (Varian) coupled to a double mass spectrometer in tandem (Varian 320 MS-MS^3^). The mobile phase comprised solvent A (0.05% formic acid) and solvent B (0.05% formic acid in methanol) as follows: 85% solvent A for 1 min 30 s (flow rate 0.4 ml/min), followed by 3 min in which solvent B increased till 98% (0.2 ml/min) which continued for 5 min 30 s with the same flow rate, followed by 2 min 30 s with increased flow rate (0.4 ml/min), subsequently returning to 85% solvent A in 1 min, conditions that were kept till the end of the run, in total 15 min. Compounds were detected in the electrospray ionization negative mode. Molecular ions [M-H]^-^ at *m*/*z* 137 and 209 and 141 and 213 generated from endogenous SA and JA and their internal standards, respectively, were fragmented under 12 V collision energy. The ratios of ion intensities of their respective daughter ions, *m*/*z* 93 and 97 and *m*/*z* 59 and 63, were used to quantify endogenous SA and JA, respectively.

### QUANTITATIVE RT-PCR

For analysis of transcript levels, total RNA was isolated using Trizol from 10 infiltrated leaves, harvested from 10 different plants, in three independent experiments and treated with TurBo DNA-free (Ambion^4^) to remove DNA. cDNA was synthesized from 1 μg of total RNA using M-MuLV reverse transcriptase (Fermentas^5^), as described by the manufacturer, in a 20-μl reaction that was diluted to 50 μl prior to using it for the real-time PCR. This was performed in a 20-μl volume containing 2 μl of cDNA, 0.4 pmol of specific primer sets for each gene and 10 μl of iTaq^TM^ SYBR Green Supermix with ROX (Bio-Rad^6^). PCR conditions were as follows: 95°C for 2 min 30 s (first cycle), 95°C for 15 s and 60°C for 30 s (40 cycles). To ensure amplification of a single product during the qRT-PCR reactions, a dissociation protocol was performed in which samples were slowly heated from 55 to 95°C. qRT-PCR was performed using the ABI Prism 7000 real-time PCR detection system (Applied Biosystems) and the data were collected using software (ABI 7000 SDS version 1) provided by the supplier. Transcript levels were normalized to the levels of the *SAND* gene (At2g28390; Hong et al., 2010) and quantification was performed as described in previous work (Pfaffl, 2001). Primer sequences were as reported in (Anderson and Badruzsaufari, 2004; Czechowski et al., 2005; Park et al., 2010) for *PR1*, *VSP2*, *LEC*, and *SAND*, respectively.

### TRYPAN BLUE AND ANILINE BLUE STAINING

Trypan blue staining solution was prepared by adding trypan blue to lactophenol (10 ml lactic acid, 10 ml glycerol, 10 ml phenol, and 10 ml distilled water) to a concentration of 2.5 mg/ml. Two volumes of ethanol were added to the trypan blue–lactophenol solution. To visualize plant cell death, mock and DC3000 infected leaf tissues were placed in plates containing staining solution and heated in a microwave at intervals for 1 min. The plates were incubated for 2 h at room temperature, followed by destaining (three times) in chloral hydrate (2.5 g/ml). The leaf tissues were mounted in 70% glycerol for observations with a microscope. For detection of callose deposition, leaves were incubated for at least 24 h in 96% ethanol until all tissues were transparent and stained in 0.01% aniline blue in 0.15 M K_2_HPO_4_ (pH 8.5). Leaf tissues were incubated for 1.5–3 h, mounted on slides, and observed under an epifluorescence microscope (AF6000) with UV filter (excitation filter: BP 470/40 nm; emission filter: BP 525/50 nm).

### CALLOSE QUANTIFICATION

Callose was quantified from digital photographs as the number of white pixels, covering the whole leaf material, using Photoshop CS7 software. Contrast settings of photographs were adjusted to obtain an optimal separation of the callose signal from the background signal. Callose was selected automatically, using the “Color Range” tool. In cases in which the contrast settings resulted in significant loss of callose signal, due to high autofluorescence of vasculature tissue, callose was selected manually, using the “Magic Wand” tool of Photoshop CS7. Relative callose intensities were quantified as the number of fluorescent callose-corresponding pixels relative to the total number of pixels covering plant material (Luna et al., 2011).

### *E*-2-HEXENAL TREATMENT

Plants were grown for 3 weeks under the conditions mentioned above before being exposed to volatiles. For the volatile treatment, 10 plants in single pots were placed into airtight glass desiccators (22 l). *E*-2-hexenal was diluted in methanol, and applied to a sterile cotton swab, placed in an Erlenmeyer flask, between the plants in the desiccators to give a final concentration of 3 μM. For the control treatment, only methanol was applied. Plants were incubated in the desiccators for 24 h and subsequently taken out to be placed under the growth conditions described above for 1 h, prior to infiltration with bacteria or mock solution as mentioned above. *E*-2-hexenal was purchased from Sigma-Aldrich.

## RESULTS

### *hpl1* INFLUENCES SUSCEPTIBILITY TO *Pseudomonas syringae* pv. *tomato* (DC3000)

**FIGURE 1:**
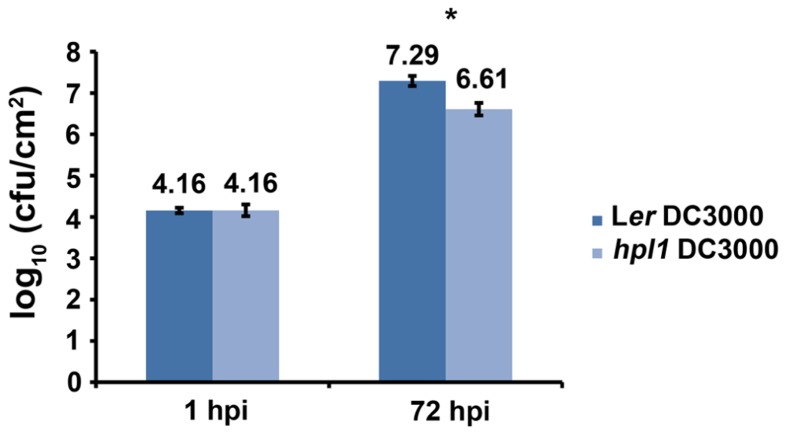
***HPL* influences bacterial growth**. Bacterial populations of DC3000 in infected L*er* and *hpl1* leaves 1 hours post infection (hpi) and 72 hpi. Values are the mean of 27 sets of two leaf disks from 20 plants. Error bars represent standard error. Bars annotated with an asterisk indicate significant differences among 72 hpi samples (*P* < 0.05, according to Student’s *t*-test analysis). The data presented are from a representative experiment that was repeated four times with similar results.

In order to determine whether the ability to synthesize GLVs had an effect on *Arabidopsis* susceptibility to pathogenic bacteria, we compared Landsberg *erecta* (*HPL*, L*er*) and an introgression line between Col-0 and L*er* that can synthesize only trace amounts of GLVs, *hpl1* (Shiojiri et al., 2012), for the susceptibility to *Pseudomonas syringae* pv. *tomato* DC3000. To ensure infection throughout the entire leaf, we used the syringe infiltration method since it overcomes stomatal defenses and maximizes the number of responding cells (de Torres Zabala et al., 2009), and bacterial populations were determined 72 hpi (hours post-infection). **Figure 1** shows that DC3000 populations were lower in the *hpl1* line. The difference measured in bacterial population between L*er* and *hpl1* (~4.6-fold) was statistically significant (*t*-test *P* < 0.05). This indicates that the *hpl1* line is less susceptible to DC3000 than L*er*.

### *hpl1* INFLUENCES JA AND SA LEVELS DURING THE INFECTION WITH DC3000

**FIGURE 2:**
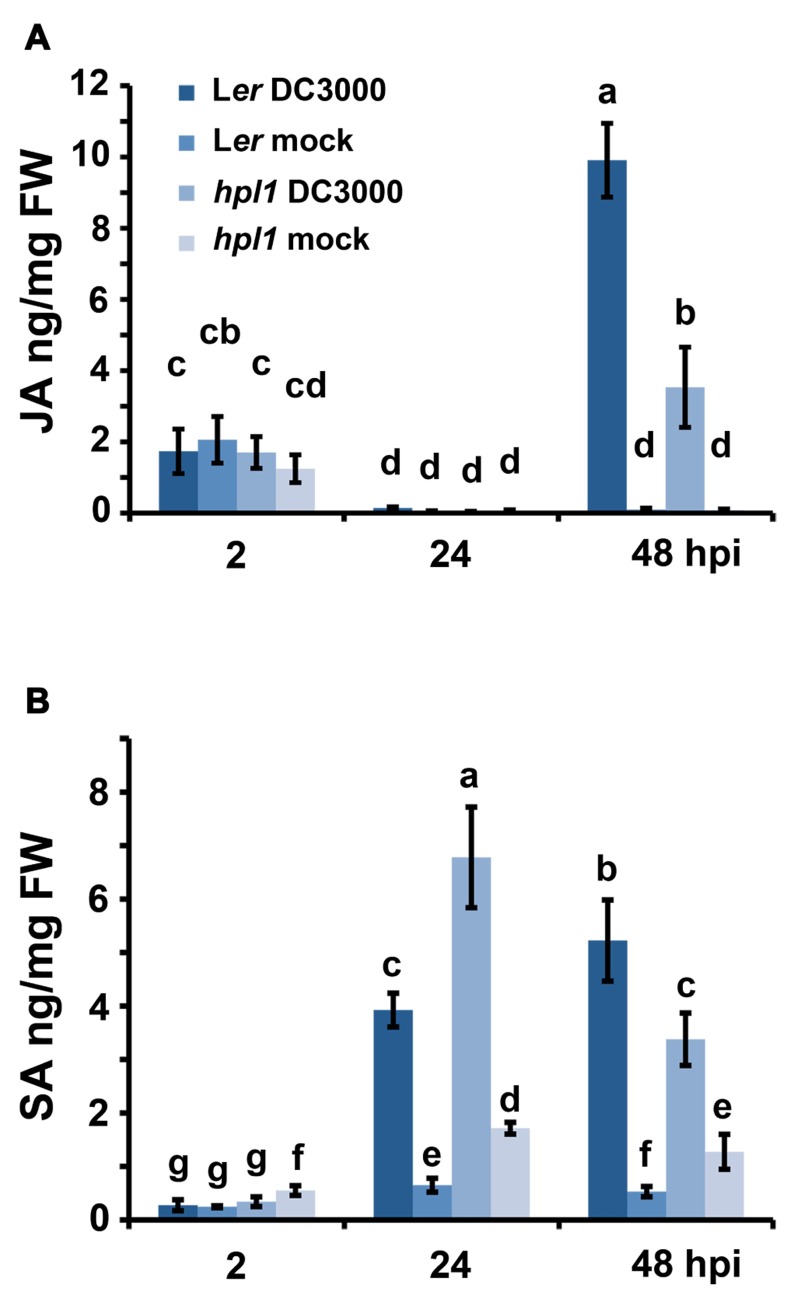
**DC3000 infection results in higher JA levels in L*er* plants and higher SA levels in *hpl1* plants**. **(A)** JA levels in L*er* and *hpl1* infected with DC3000 at 2, 24, and 48 hpi; **(B)** SA levels in L*er* and *hpl1* plants infected with DC3000 at 2, 24, and 48 hpi. In both cases, the hormone levels in the 10 mM MgSO_4_ (mock) infiltrated plants are also shown. For each timepoint and genotype, nine leaves were harvested, in pools of three from mock-infiltrated or bacteria-infiltrated plants and used for plant hormone quantification. Bars represent the mean of two independent experiments. Error bars represent standard error. Bars annotated with different letters indicate statistically different hormone levels [*P* < 0.05, according to analysis of variance (ANOVA), followed by a least significant difference (LSD) *post hoc* test].

It is well known that the balance between JA and SA is crucial for the interaction that will be established between a pathogen and its host (Spoel and Dong, 2008; Grant and Jones, 2009; Pieterse et al., 2009). We therefore monitored the changes in JA and SA in L*er* and the *hpl1* plants, prior to the bacterial population measurement, at 2, 24, and 48 hpi. As shown in **Figure 2A**, the levels of JA were up at 2 hpi in all treatments, most likely because of the mechanical damage caused by the inoculation with the syringe. At 24 hpi, this wound response was reset, as JA levels were very low, comparable to the mock inoculation. The situation changed at 48 hpi when JA levels increased in DC3000 infested leaves, in L*er* approximately threefold higher than in *hpl1*. SA levels (**Figure 2B**) changed already at 24 hpi, with levels being approximately 1.7-fold higher in *hpl1* than in L*er*, suggesting that SA-related defenses are activated earlier in *hpl1*. In L*er*, the SA levels were higher than in *hpl1* at 48 hpi suggesting that these defenses are mounted later in L*er*.

### JA MARKER GENES ARE LESS INDUCED IN *hpl1* THAN L*er* WHEN INFECTED WITH DC3000

**FIGURE 3:**
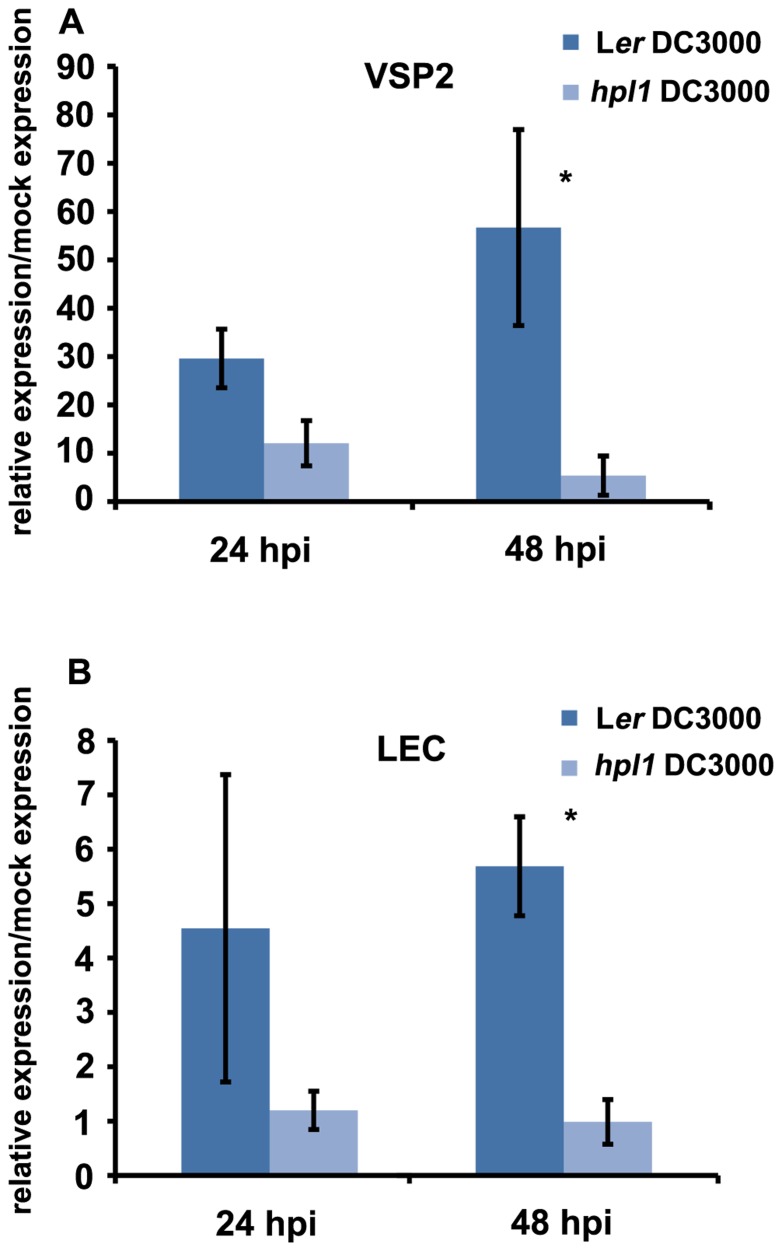
**JA-dependent gene expression is higher in infected L*er* plants**. **(A)** VSP2 transcript levels and **(B)** LEC transcript levels were measured by qRT-PCR in L*er* and *hpl1* infected with DC3000 at 24 and 48 hpi and normalized for SAND transcript levels. Bars represent the ratio between the transcript levels in infected and mock samples. Three infected or mock infiltrated leaves were harvested from three different plants and pooled for RNA isolation. Bars represent the mean of three independent experiments. Error bars represent standard error. Bars annotated with asterisk indicate significant differences among samples (*P* < 0.05, according to *t*-test analysis).

**Figure A1:**
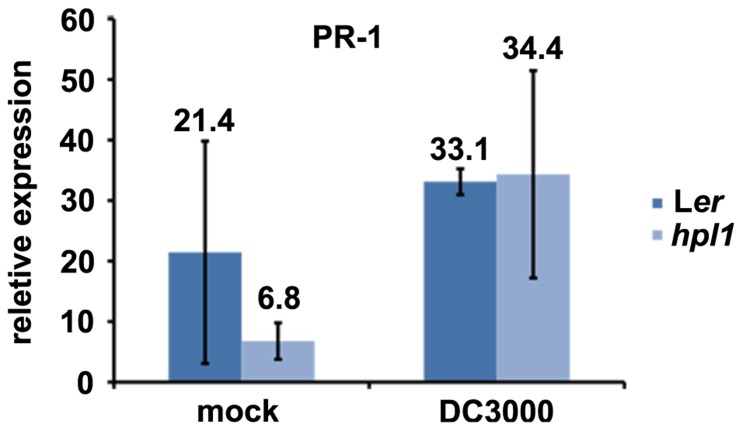
***PR-1* expression is equally induced in L*er* and *hpl1***. *PR-1* transcript levels were measured by qRT-PCR in L*er* and *hpl1* infected with DC3000 48 hpi and normalized for SAND transcript levels. Error bars represent standard error.

In order to determine whether the differences in hormone levels had an effect on the expression of relevant marker genes in our system, we performed qRT-PCR for genes downstream of JA and SA. We chose *VSP2* and *LEC* for JA (Potter et al., 1993; Penninckx et al., 1998; Thomma et al., 1998; Liu et al., 2005; Pré et al., 2008) and *PR-1* for SA (Bowling et al., 1997; Clarke et al., 2001). *PR1* expression was clearly induced by DC3000 at 48 hpi, however, to similar levels in L*er* and *hpl1* plants (**Figure A1** in Appendix). In contrast, transcript levels of both *VSP2* and *LEC* at 48 hpi (and 24 hpi) were much lower in *hpl1* than in L*er* (**Figures 3A,B**). This result is consistent with the observed lower JA levels in *hpl1* at 48 hpi (**Figure 2A**).

### LER (*HPL*) and *hpl1* DIFFER IN THE NUMBER OF DEAD CELLS AND IN CALLOSE DEPOSITION

**FIGURE 4:**
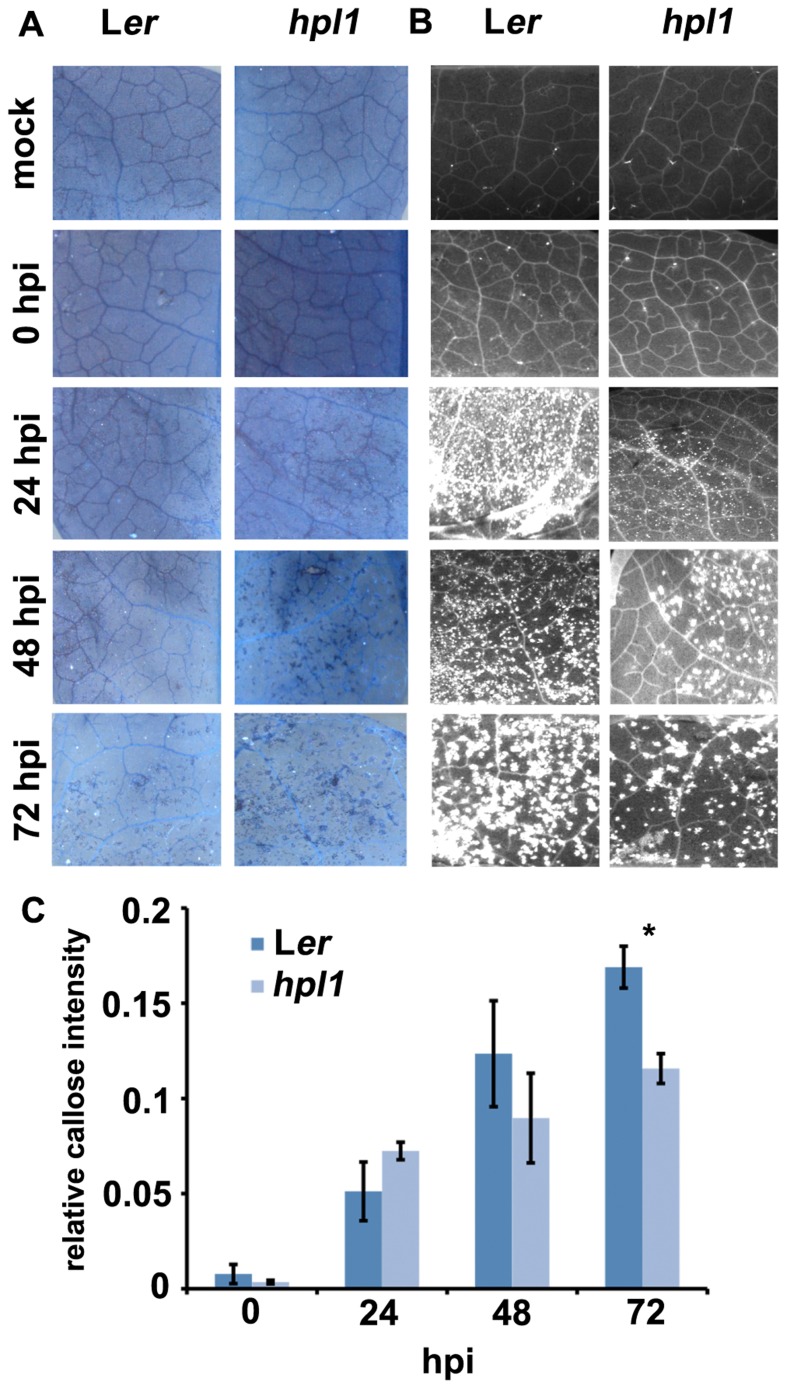
**Dead cells and callose deposition are different in L*er* and *hpl1***. **(A)** Trypan blue staining showing small clusters of dead cells in *hpl1* but not in L*er* leaves infected with DC3000. **(B)** Aniline blue stained leaf tissues observed under UV illumination showing earlier and higher callose deposition in L*er* than in *hpl1* leaves infected with DC3000. **(C)** Relative callose intensity. Bars represent the mean of three different experiments. Error bars represent standard error. Bars annotated with an asterisk indicate a significant difference among samples (*P* < 0.05, according to *t*-test analysis).

To investigate further the differences between L*er* and *hpl1* in mounting plant defense responses, we decided to look at the appearance of dead cells and callose deposition. Dead cells are indicative of programed cell death (or the hypersensitive response, HR) and enhanced resistance, usually occurring when an pathogenic effector is recognized by the host (Alfano and Collmer, 1996), whereas callose is typically triggered by conserved pathogen-associated molecular patterns (PAMPs), such as flagellin, at the sites of infection during the relatively early stages of pathogen invasion (Brown et al., 1998; Gómez-Gómez et al., 1999; Jones and Dangl, 2006). Dead cells appeared earlier and more frequently in the more resistant *hpl1* while callose deposition occurred earlier and more abundantly in the more susceptible L*er* (**Figures 4A–C**). Dead cells appeared at day 2 in *hpl1*, whereas in L*er* they were not present at all, even at day 3. L*er* started to deposit callose massively at day 1, while much less papillae at this time could be observed in *hpl1*. Moreover, even at later stages of infection, at days 2 and 3, L*er* showed more callose deposition than *hpl1*.

### *E*-2-HEXENAL TREATMENT INCREASES SUSCEPTIBILITY TO DC3000

**FIGURE 5:**
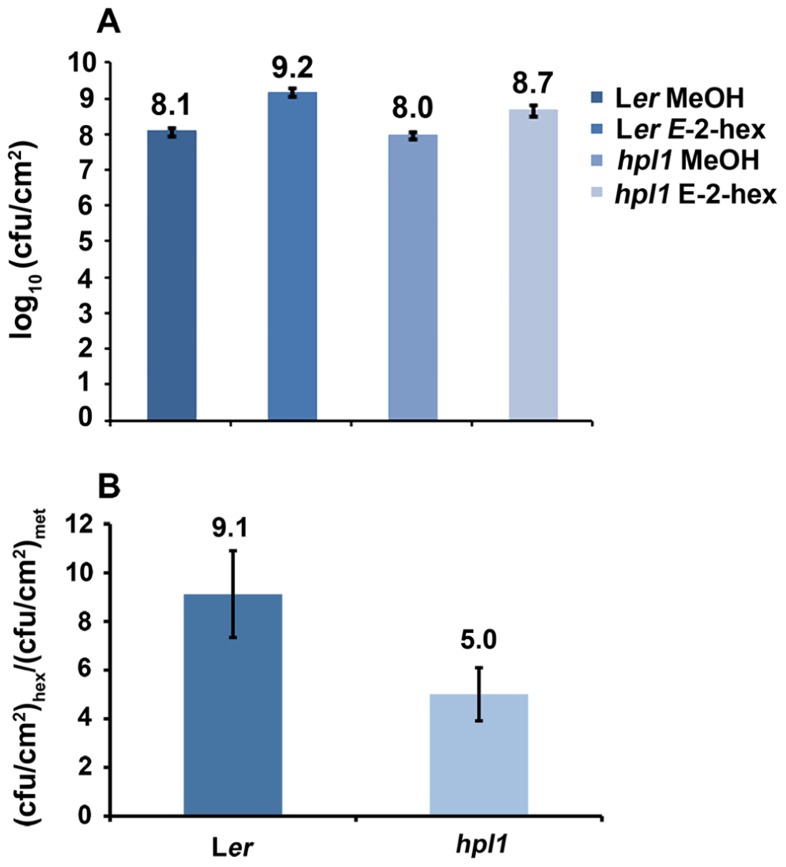
***E*-2-hexenal pre-treatment increases susceptibility to DC3000**. **(A)** DC3000 populations in L*er* and *hpl1* pre-treated with 3 μM *E*-2-hexenal or methanol were measured 72 hpi. Values are the mean of 16 sets of two leaf disks from 12 plants. Error bars represent standard error. The data presented are from a representative experiment that was repeated four times with similar results. All pre-treatments with *E*-2-hexenal were significantly different from the control treatment (*P* < 0.05, according to Student’s *t*-test analysis). **(B)** Bars represent the ratio between cfu/cm^2^ with *E*-2-hexenal pre-treatment and cfu/cm^2^ with methanol pre-treatment (control). Values are the mean of three independent experiments. Error bars represent standard error.

**Figure A2:**
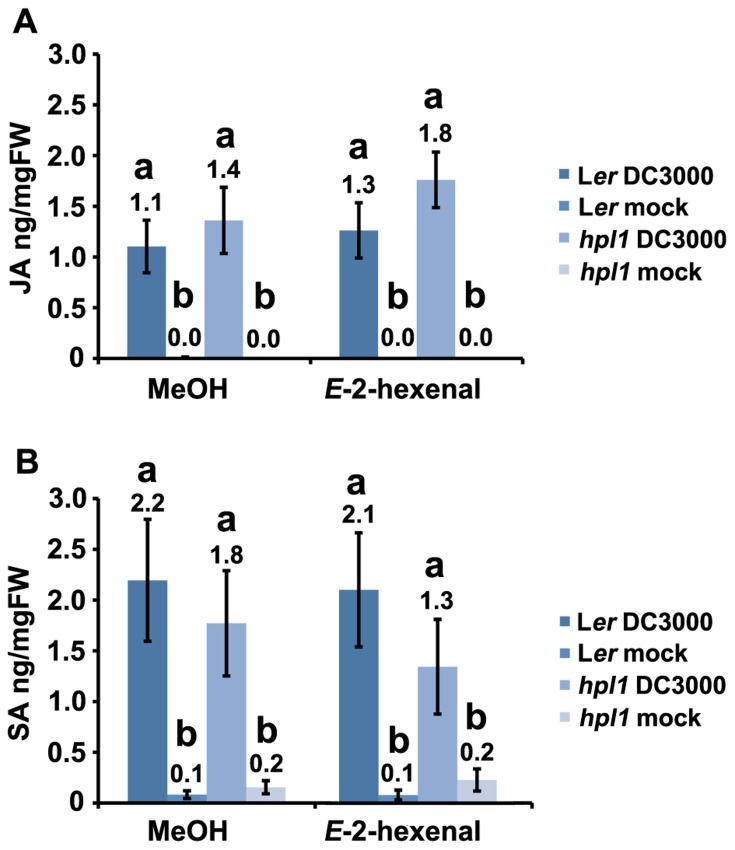
***E*-2-hexenal does not induce changes in JA and SA levels in L*er* and *hpl1* plants infected with DC3000**. **(A)** JA levels in L*er* and *hpl1* plants pre-treated with *E*-2-hexenal or MeOH and subsequently infected with DC3000 (48 hpi); **(B)** SA levels in L*er* and *hpl1* plants pre-treated with *E*-2-hexenal or MeOH and subsequently infected with DC3000 (24 hpi). In both cases the hormone levels in the 10 mM MgSO_4_ (mock) infiltrated plants are also shown. Nine leaves were harvested, in pools of three from mock-infiltrated or bacteria-infiltrated plants at specified timepoints and used for plant hormone quantification. Bars represent the mean of three independent experiments. Error bars represent standard error. Bars annotated with different letters indicate statistically different hormone levels (*P* <0.05, according to ANOVA, followed by a LSD *post hoc* test).

Since *hpl1* is unable to produce GLVs, we addressed the question whether application of GLVs would restore its susceptibility to DC3000 comparable to L*er*. We chose to use the C6-aldehyde *E*-2-hexenal, one of the most active GLVs, and treated *hpl1* and L*er* plants with 3 μM aerial *E*-2-hexenal or with the carrier methanol (MeOH) for the control treatment. **Figure 5A** shows that the treatment with the C_6_-aldehyde turned both *hpl1* and L*er* more susceptible to DC3000, as bacterial populations increased about five- and ninefold, respectively, in the *E*-2-hexenal pre-treated leaves compared to the control pre-treatment (**Figure 5B**). Additionally, we measured JA and SA levels in L*er* and *hpl1* plants infected with DC3000 after pre-treatment with *E*-2-hexenal or MeOH. Although JA and SA levels increased 48 hpi after DC3000 infection, no significant differences in hormone levels were detected between the *E*-2-hexenal and the control treatment or between L*er* and *hpl1* (**Figure A2** in Appendix).

### THE EFFECT OF *E*-2-HEXENAL ON BACTERIAL GROWTH ACTS VIA ORA59.

**FIGURE 6:**
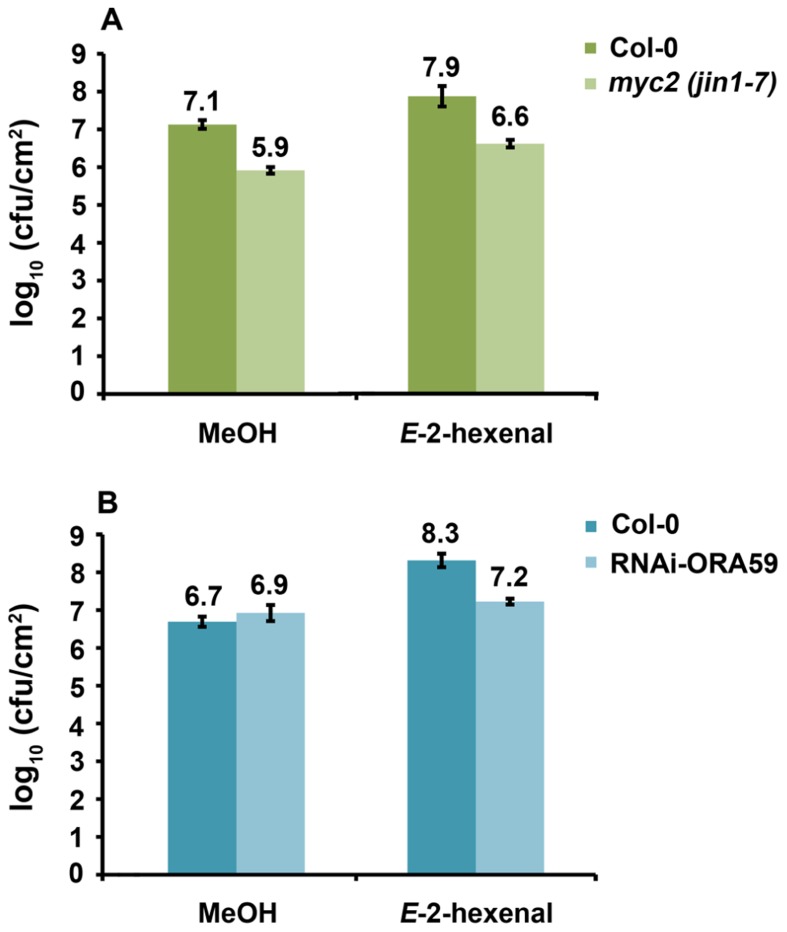
**Reduction of *ORA59* expression influences *E*-2-hexenal effect on bacterial growth**. **(A)** Bacterial populations of DC3000 in inoculated *myc2* (*jin1-7*) and Col-0 leaves 72 hpi. Plants were pre-treated 24 h with 3 μM *E*-2-hexenal or methanol. **(B)** Bacterial populations of DC3000 in inoculated RNAi-ORA59 and 35S:GUS plants at 72 hpi. Plants were pre-treated with 3 μM *E*-2-hexenal or methanol for 24 h. Values are the mean of 24 sets of two leaf disks from 20 plants. Error bars represent standard error. All pre-treatments with *E*-2-hexenal were significantly different from the control treatment (*P* < 0.05, according to Student’s *t*-test analysis), except for RNAi-ORA59. The data presented are from a representative experiment that was repeated three times with similar results.

Since a functional HPL leads to higher susceptibility and higher JA levels upon DC3000 infection and *E*-2-hexenal pre-treatment increased susceptibility of *Arabidopsis* to DC3000 we sought to elucidate part of the signaling pathways involved, by testing if *Arabidopsis* mutants in the JA-signaling pathway were still more susceptible to DC3000 after treatment with *E*-2-hexenal. We chose to analyze MYC2 and ORA59 impaired lines since these are the main players in regulating JA-dependent responses and are located in two different branches of the JA-signaling pathway (Lorenzo et al., 2003, 2004; Anderson and Badruzsaufari, 2004; Dombrecht et al., 2007; Oñate-Sánchez et al., 2007; Kazan and Manners, 2008; Pré et al., 2008). As shown in **Figure 6A**, *myc2* (*jin1-7*) plants were more resistant to DC3000 as has been reported (Fernández-Calvo et al., 2011). Moreover, *myc2* as well as wild-type plants showed increased susceptibility to DC3000 when pre-treated with *E*-2-hexenal, seemingly excluding a role for MYC2 in mediating this phenomenon. In contrast, the same assay performed on RNAi-ORA59 plants (Pré et al., 2008) showed that the bacterial populations increased significantly less in the ORA59 silenced plants compared to the corresponding control line after *E*-2-hexenal treatment (**Figure 6B**). This indicates an involvement of ORA59 in this response to *E*-2-hexenal.

### THE *E*-2-HEXENAL EFFECT IS CORONATINE DEPENDENT

**FIGURE 7:**
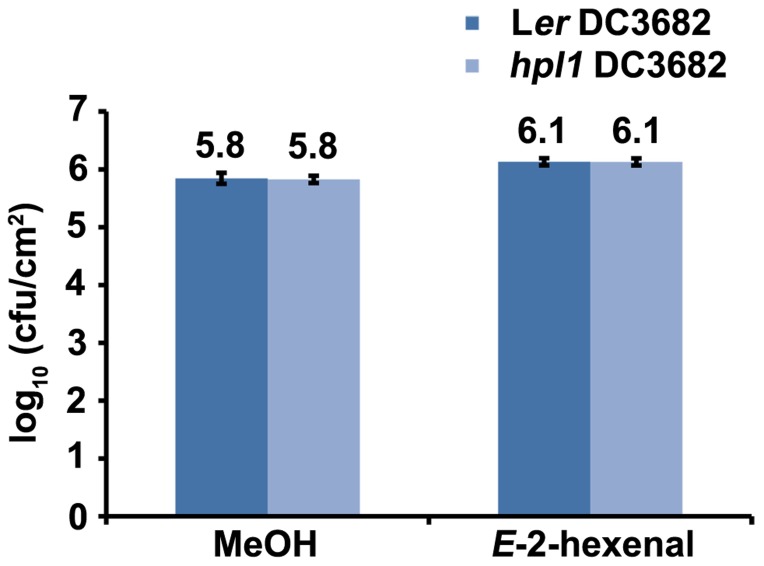
**The effect of *E*-2-hexenal is partially dependent on coronatine**. Bacterial populations of the *cor* mutant (DC3682) in inoculated L*er* and *hpl1* leaves at 72 hpi. Plants were pre-treated 24 h with 3 μM *E*-2-hexenal or methanol. Values are the mean of 24 sets of two leaf disks from 20 plants. Error bars represent standard error. All pre-treatments with *E*-2-hexenal were significantly different from the control treatment (*P* < 0.05, according to Student’s *t*-test analysis). The data presented are from a representative experiment that was repeated three times with similar results.

*Pseudomonas syringae* pv. *tomato* strain DC3000 synthesizes COR (Mitchell, 1982), a phytotoxin that mimics JA-Ile (Thines et al., 2007; Yan et al., 2009), in order to antagonize the SA-dependent defenses (Brooks et al., 2005; Glazebrook, 2005). Therefore, we also determined whether the production of COR was necessary for DC3000 to proliferate more in *E*-2-hexenal treated plants. For this, L*er* and *hpl1* plants were infected with the *Pseudomonas syringae* mutant strain DC3682 (Ma et al., 1991), that is unable to produce COR, after pre-treatment with *E*-2-hexenal or methanol. **Figure 7** shows that the bacterial populations of the *cor* mutant were only slightly, but significantly, higher in L*er* or *hpl1* plants treated with *E*-2-hexenal compared to the control plants, but that this increase was much lower than for DC3000 (**Figure 1**). Thus COR seems to be necessary for DC3000 to benefit from the *E*-2-hexenal treatment.

## DISCUSSION

Green leaf volatiles have received considerable attention for their ability to induce direct and indirect defense responses in plants and can be considered important players in the already complex network regulated during biotic stress. However the mechanisms by which GLVs influence pathogenesis, and the signaling pathways involved in these responses, are not well known. To address this, we used L*er* and its *Arabidopsis* introgression line, *hpl1*, lacking GLV synthesis, and analyzed their response during infection with the bacterial pathogen *Pseudomonas syringae* pv. *tomato* (DC3000). DC3000 was chosen because in some plant species such as lima bean and tobacco, infection triggers *E*-2-hexenal emission (Croft et al., 1993; Heiden et al., 2003). We hypothesized that *hpl1* plants would be more susceptible to DC3000 since there is evidence that GLVs and *E*-2-hexenal have antimicrobial properties (Prost et al., 2005), induce defense-related genes or biosynthesis of defense-related secondary metabolites (Bate and Rothstein, 1998; Arimura et al., 2001; Gomi et al., 2003; Weber et al., 2004; Farag et al., 2005; Kishimoto et al., 2005, 2006; Paschold et al., 2006), and increase resistance against *B. cinerea* (Kishimoto et al., 2005). However, we found the opposite result: plants impaired in GLV production were more resistant to DC3000 (**Figure 1**). A similar result was very recently shown in rice where the mutant Os*hpl3*, not able to synthesize GLVs, was more resistant to *Xanthomonas oryzae* pv. *oryzae* (Tong et al., 2012).

Subsequently, we investigated some of the mechanisms underlying this result by analyzing the levels of SA and JA since it is well known that these phytohormones and their antagonism are crucial for the development of pathogenesis in *Arabidopsis* (Spoel and Dong, 2008; Grant and Jones, 2009; Pieterse et al., 2009). Hormone measurements clearly showed that JA levels were much lower in *hpl1* than in L*er* (**Figure 2A**). Conversely, *hpl1* showed an earlier induction of SA than L*er* (**Figure 2B**). These data suggest that a non-functional *HPL* gene influences the JA-branch of the oxylipin pathway, leading to lower production of JA when *Arabidopsis* is challenged with *Pseudomonas*. Thus, this is not related to substrate competition as previously shown in *Arabidopsis* where ectopic expression of *HPL* led to lower JA levels upon wounding (Chehab et al., 2006). Reduction of *HPL* expression in rice and *N. attenuata* also influenced JA levels but differently: Os*hpl3* and *asHPL1* had increased JA levels (Halitschke et al., 2004; Tong et al., 2012), in *N. attenuata* probably due to crosstalk between the GLV and JA pathway (Allmann et al., 2010).

Since JA-signaling downstream of COI1 occurs via two different branches, regulated by MYC2 or ORA59, we used markers for both branches to study their activation after DC3000 infection. *LEC*, a lectin-like gene, was used for the ORA59 pathway since it is induced by methyl-jasmonate and upon ORA59 overexpression (Schenk et al., 2000; Pré et al., 2008), while *VSP2* was used for the MYC2 pathway (Abe et al., 2003; Dombrecht et al., 2007). Both *VSP2* and *LEC* transcript levels were much lower in *hpl1* than in L*er* (**Figures 3A,B**) concurrent with the lower JA levels. Thus DC3000 activates in L*er*, with an active HPL unlike Col-0 (Duan et al., 2005), with which most DC3000 experiments are carried out, both branches of the JA-signaling pathway and antagonistic control of these distinct branches of the JA pathway (Verhage et al., 2011) is apparently minor. Transcript levels of the SA-marker *PR-1* were higher upon DC3000 infection, similarly in *hpl1* and L*er* (**Figure A1** in Appendix), probably because the differences in SA levels between the two genotypes were not big enough to cause a difference. Thus it seems that the lower JA levels in *hpl1* plants leads to less activation of the JA-signaling pathways and renders them less susceptible to DC3000.

A hallmark of basal plant defenses to pathogen infection is the deposition of callose. PAMP-induced callose deposition has recently been defined with essential roles for the DC3000 type III effector HopM1 and COR suppressing callose deposition, the latter being, interestingly, partly COI1-independent (Geng et al., 2012). Our results showed that in *hpl1*, although with smaller bacterial populations than in L*er*, clearly less callose was deposited (**Figures 4B,C**). Ethylene (ET) signaling it is crucial for callose deposition in response to flagellin (Clay et al., 2009). It is possible that this ET signaling is less activated in *hpl1*, leading to less callose deposition. Support for this comes from our complementation studies with the *hpl1* mutant, a response that is largely dependent on ORA59, a TF that integrates JA and ET signaling (**Figure 6B**). Perhaps related to this is the fact that DC3000 is apparently less effective in preventing cell death in *hpl1* than in L*er* (**Figure 4A**), with fewer living cells producing less callose. DC3000 apparently triggers in *hpl1* a higher rate of cell death, which is related to higher resistance (Jones and Dangl, 2006).

With the aim to overcome the *hpl1* phenotype in response to DC3000 infection, we decided to treat these, and L*er*, plants with *E*-2-hexenal. The pre-treatment with 3 μM *E*-2-hexenal for 24 h prior to DC3000 infection made *hpl1* plants considerably more susceptible to DC3000 (**Figures 5A,B**). The increase in bacterial populations was about ninefold in L*er* and fivefold in *hpl1* plants. Thus L*er* plants remained more susceptible to DC3000 than *hpl1* plants, most likely due to the functional HPL. Due to its high reactivity for being a reactive electrophile species (RES), *E*-2-hexenal, either induced during the HR or exogenously applied, can undergo conjugation to glutathione (GSH), leading to the formation of *E*-2-hexenal-GSH adducts in the form of 1-hexanol-3-GSH (Davoine et al., 2006; Mirabella et al., 2008). Conjugation to GSH is a well-known mechanism to inactivate reactive molecules (Coleman et al., 1997). Additionally, conjugation to cellular proteins has been reported to occur for several RES, including *E*-2-hexenal (Davoine et al., 2006; Myung et al., 2007; Dueckershoff et al., 2008; Mueller et al., 2008; Yamauchi et al., 2008). Therefore, we cannot exclude the possibility that, through conjugation, *E*-2-hexenal affects the function of proteins involved in the plant defense responses to DC3000, making *Arabidopsis* more susceptible to this pathogen. A similar effect has been reported for syringolin, a toxin with an unsaturated α,β carbonyl moiety, that makes it a RES, produced by, e.g., *Pseudomonas syringae* pv. *syringae*. This toxin specifically inhibits the proteasome in order to suppress host defenses (Groll et al., 2008; Schellenberg et al., 2010).

Analyses of phytohormone levels after treatment of *E*-2-hexenal and DC3000 infection showed that there were no statistically significant differences in SA and JA levels between control and treatment (**Figure A2** in Appendix). So far only in monocots (maize) an increase in JA has been measured after a GLV treatment (Engelberth et al., 2004; Engelberth, 2011). In the JA-signaling pathway COI1 plays a central role and mutants in this gene are blocked in almost all JA responses (Feng et al., 2003; Devoto et al., 2005; Wang et al., 2008). Downstream of COI1, different TFs regulate specific JA-dependent responses: MYC2 and ORA59 are the main players involved. The MYC2-dependent branch is associated with wound response, responses against herbivores and is also regulated by abscisic acid (ABA; Lorenzo et al., 2003). This basic helix-loop-helix (bHLH) transcription factor regulates a large number of JA-responsive genes (Dombrecht et al., 2007), among which VEGETATIVE STORAGE PROTEIN2 (VSP2; Liu et al., 2005). In the other branch, ORA59 integrates JA and ET signaling (Pré et al., 2008). Interestingly, in spite of the absence of difference in JA and SA levels, the higher susceptibility of *Arabidopsis* plants to DC3000 after *E*-2-hexenal treatment was dependent on ORA59. The DC3000 bacterial populations increased only slightly in ir-ORA59 plants after *E*-2-hexenal treatment as compared to control (35S-GUS) plants (**Figure 6B**), indicating the relevance of JA signaling, and perhaps ET signaling. A role for MYC2 in this process was excluded based on the fact that *myc2* mutants still responded to exogenous *E*-2-hexenal treatment (**Figure 6A**).

From the bacterial side we investigated whether the production of COR was necessary to benefit from the *E*-2-hexenal treatment. For this we employed *cor*, a COR-deficient strain, to infect plants, after the *E*-2-hexenal or control treatment. The result showed that there was a small but significant increase in bacterial populations of the *cor* strain after the *E*-2-hexenal treatment (**Figure 7**). Nevertheless this difference was much smaller than for DC3000, suggesting that COR is necessary for DC3000 to fully benefit from GLVs.

Our data show that a functional *HPL* in *Arabidopsis* promotes susceptibility to DC3000. This effect is partially mediated by ORA59 in the plant and by COR in the bacteria.

The question remains how DC3000 precisely exploits HPL or its products, GLVs or the C_12_ compounds that are also formed in the HPL pathway (Kallenbach et al., 2011), for its benefit. Since it is clear that some herbivores can lower *HPL* transcript levels (Halitschke et al., 2004; Savchenko et al., 2012), we propose that HPL may be a target for DC3000 to employ in *Arabidopsis*, albeit to its own advantage.

## Conflict of Interest Statement

The authors declare that the research was conducted in the absence of any commercial or financial relationships that could be construed as a potential conflict of interest.

## Footnotes

1 http://www.cdnisotopes.com

2 http://www.bertin.fr

3 http://www.home.agilent.com

4 http://www.ambion.com

5 http://www.fermentas.com

6 http://www.bio-rad.com
